# Cutaneous Adnexal Cylindroma of Breast: Epithelial Immunoreactivities for GATA-3, Mammaglobin, and E-Cadherin Do Not Equate to a Mammary Ductal Neoplasm

**DOI:** 10.1155/2018/4039545

**Published:** 2018-02-13

**Authors:** A. Halima, A. M. Pannunzio, E. M. Erstine, J. S. Ko, W. F. Bergfeld, R. M. Malaya, M. B. Frankel, B. C. Calhoun, C. D. Sturgis

**Affiliations:** ^1^RJ Tomsich Pathology and Laboratory Medicine Institute, Cleveland Clinic, Cleveland, OH, USA; ^2^General Surgery, Cleveland Clinic, Cleveland, OH, USA; ^3^Diagnostic Radiology, Cleveland Clinic, Cleveland, OH, USA

## Abstract

Cylindromas are benign epithelial neoplasms derived from cutaneous eccrine adnexal structures. These tumors are most commonly encountered on the head, neck, and scalp of older women. In rare instances, solitary cylindromas may arise at other body sites. In the current case, a cylindroma of the skin of the breast was diagnosed by complete excision. Immunohistochemical studies confirmed the tumor cells to be immunoreactive with cytokeratin AE1/3, cytokeratin 5/6, cytokeratin 7, p63, and SOX10. The neoplastic cells were also noted to be immunoreactive with markers typically expected to be positive in ductal epithelium of the breast including GATA3, mammaglobin, and E-cadherin. The case emphasizes the importance of correlating clinical setting, imaging studies, patient history, and careful microscopic evaluation in arriving at an accurate diagnosis. This case also illustrates the point that not all “breast” tumors that are confirmed to be positive for GATA3, mammaglobin, and E-cadherin are derived from mammary ducts.

## 1. Introduction

Sporadic cutaneous cylindromas are most commonly encountered as solitary lesions on the neck, head, and scalp of middle-aged and elderly females, without specific racial or ethnic propensity [1–3]. Solitary cylindromas classically behave in a benign fashion. The development of most cylindromas is not related to genetic germline mutations, and such lesions typically appear as small, painless, firm, pink to red nodules [1–3]. In some instances, multiple coalescing cylindromas of larger size may cause disfigurement of the scalp and face (turban tumors) [4]. Rarely, multiple cylindromas can be encountered in familial cylindromatosis, an autosomal dominant genetic condition, or in Brook-Spiegler syndrome, a clinical setting in which multiple adnexal tumor types may manifest at younger ages and may involve other body regions such as the trunk and extremities [4–6]. Prior morphologic and immunohistochemical characterization studies have concluded that sporadic cylindromas may arise from cutaneous eccrine type adnexal glands; however, some authors have also postulated apocrine or mixed eccrine/apocrine derivations [4, 7, 8]. To our knowledge, only a few cases of sporadic cylindroma associated with the skin of the breast have been documented in the medical literature [9–17]. We herein report a unique case of a solitary cutaneous cylindroma that was clinically labeled as a breast mass, and we emphasize potential diagnostic pitfalls which could be encountered on small biopsies of cylindromas of the breast.

## 2. Case Report

**Figure 1:**
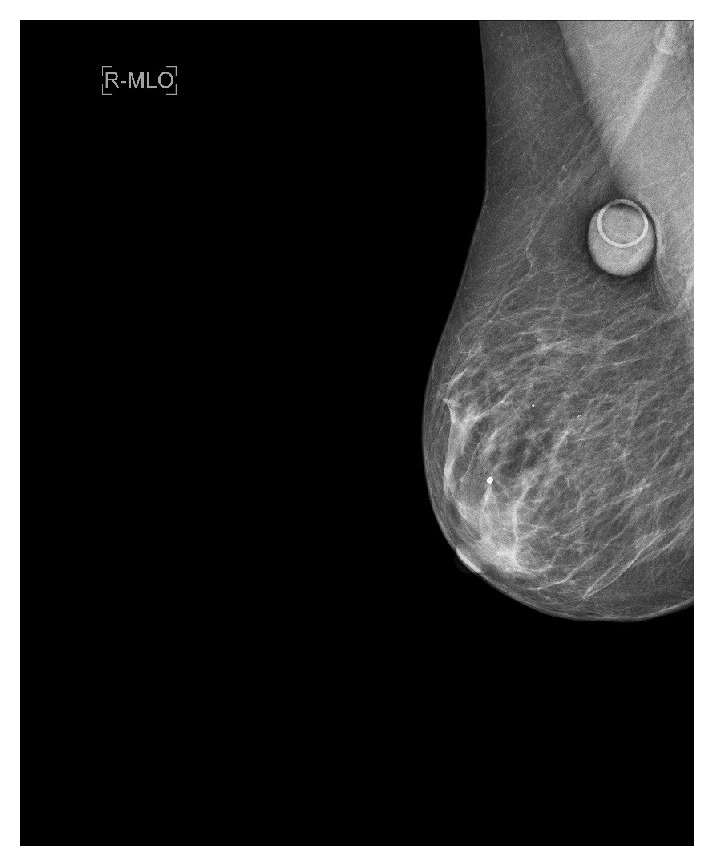
Mammogram (right mediolateral oblique view) confirming a 25 mm, well-circumscribed nodule within the substance of the breast. This lesion had been previously documented and was slowly enlarging in size over several years.

A 71-year-old Caucasian female presented with a superficially located 2.5 cm palpable mass in the right breast. This lesion had been present for at least five years and was noted to be slowly enlarging in size. The patient had a personal past history of previously resected pT2 pN1 colonic adenocarcinoma, previously resected pT1 pNX renal cell carcinoma, and previously resected basal cell carcinoma of the skin of the right clavicle. By imaging, the right breast mass was located at 12 o'clock and was described as rounded in contour without associated calcifications. A differential diagnosis of sebaceous cyst versus another well-circumscribed lesion was given (Figure 1). The lesion was completely excised one month after the time of mammography.

**Figure 2:**
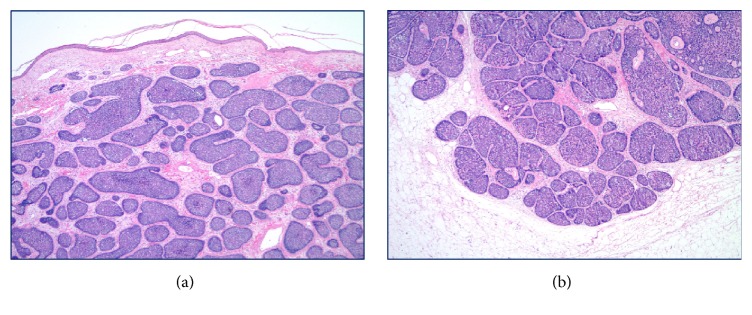
Histopathologic findings: (a) low magnification of skin surface showing intact, thinned epidermis with narrow grenz zone of uninvolved papillary dermis and subjacent epithelial proliferation of basaloid lesional cell nests arranged in a “jigsaw pattern,” H&E stain, 40x; (b) low magnification of deep edge of tumor with basaloid nests pushing into subcutaneous adipose tissue, H&E stain, 40x.

**Figure 3:**
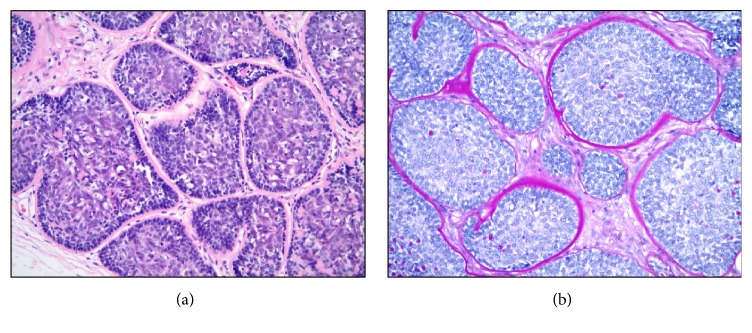
Histopathologic findings: (a) intermediate magnification of “jigsaw pattern” with biphasic cell nests showing outer somewhat palisaded small blue cells and central basaloid cells with slightly more abundant eosinophilic cytoplasm as well as fibrous stroma, H&E stain, 200x; (b) basement membrane material investing periphery of epithelial nests, PAS stain, 200x.

The specimen received in pathology was labeled as “skin, right breast, excision.” It consisted of an oriented segment of skin and underlying tissue measuring 4.6 × 2.5 × 1.9 cm. The specimen was differentially inked and serially sectioned revealing a centrally located 2.5 × 2.3 × 1.6 cm pink-tan, circumscribed, and elevated nodule that appeared to be dermally based and was grossly completely excised. The specimen was entirely submitted for microscopic analysis in ten cassettes. Histologically, an epithelial neoplasm was confirmed to be centered within the dermis and had a domed appearance. The neoplastic proliferation did not directly communicate with the epidermis, and the epidermis was intact throughout without ulceration. The tumor was comprised of nests of basaloid cells arranged in a “jigsaw” pattern. It was not encapsulated and showed a pushing border with some of the deeper nests of cells extending into the subcutaneous adipose tissue (Figure 2). The epithelial nests were separated from one another by mildly hypercellular bands of intervening spindled fibroblasts. Most of the nests of lesional epithelium appeared intimately surrounded by a layer of homogenous eosinophilic basement membrane-like material, and some nests also showed small cylindrical accumulations of similar intrinsic material. Some tumor cell nests appeared to be comprised entirely of cells with a basaloid phenotype and a suggestion of peripheral palisading, while other nests appeared somewhat morphologically biphenotypic with slightly smaller and darker peripheral myoepithelial-like cells as well as a second population of central cells with more abundant and eosinophilic cytoplasm and a tendency toward spindling or whorling (Figure 3). A histologic diagnosis of cylindroma was rendered based upon examination of routine hematoxylin and eosin (H&E) stained histologic sections and a supplemental periodic acid-Schiff (PAS) stain. The patient's surgical site healed quickly and without incident.

**Figure 4:**
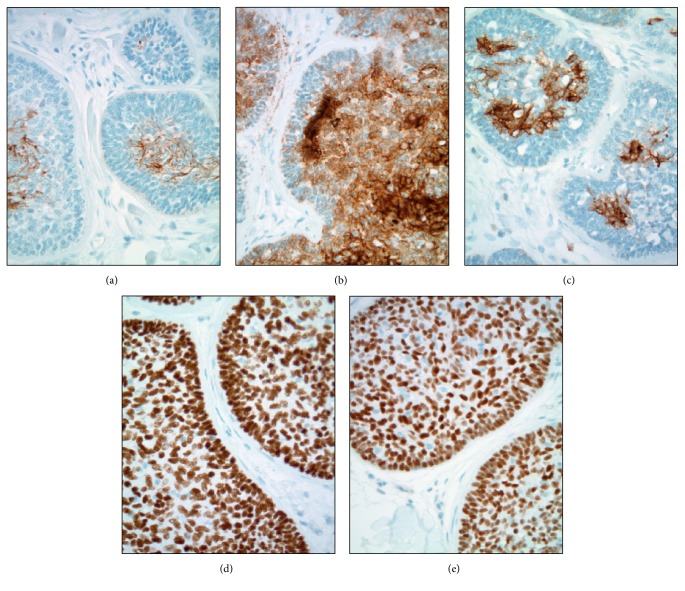
High magnification immunohistochemistry of selected cutaneous basaloid tumor markers: (a) focal membranous and cytoplasmic positivity in center of tumor cell nests with sparing of outer cells, CKAE1/3, 400x; (b) diffuse and strong cytoplasmic positivity in center of tumor cell nests with comparative sparing of myoepithelial-like edges, CK5/6, 400x; (c) patchy cytoplasmic and membranous positivity in center of tumor cell nests with sparing of outer cells, CK7, 400x; (d) diffuse and strong nuclear positivity throughout tumor cell nests, p63, 400x; (e) diffuse nuclear positivity of moderate to strong intensity throughout tumor cell nests, SOX10, 400x.

**Figure 5:**
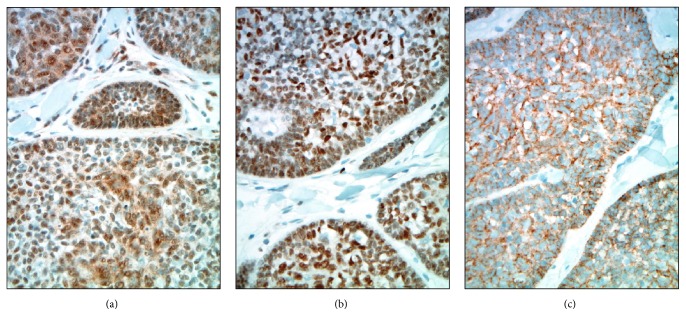
High magnification immunohistochemistry of selected breast tumor markers: (a) diffuse nuclear and cytoplasmic positivity of moderate intensity, mammaglobin, 400x; (b) diffuse and strong nuclear and focal cytoplasmic positivity throughout tumor cell groups, GATA3, 400x; (c) diffuse and moderately intense cytoplasmic positivity throughout tumor cell groups, E-cadherin, 400x.

For reasons of academic interest, a battery of immunohistochemical studies was pursued on a representative formalin-fixed and paraffin-embedded cylindroma tissue block. All of these tests were performed using standard protocols in the Cleveland Clinic clinical immunohistochemistry laboratory. These studies showed the neoplasm to be immunoreactive (positive) with various cytokeratins including AE1/3, CK5/6, and CK7. The AE1/3 and CK7 patterns of immunoreactivity were similar with staining of the center of some basaloid tumor cell nests in a mixed membranous and cytoplasmic pattern of positivity (Figure 4). The CK5/6 slide showed diffuse cytoplasmic positivity in the lesional cells with some sparing of the extreme peripheral palisading myoepithelial-like layers. The tumor cells were also noted to show diffuse nuclear immunoreactivity with p63 and SOX10 (Figure 4). In addition, mammaglobin, GATA3, and E-cadherin were found to be immunoreactive in the majority of tumor cells (Figure 5). Other immunohistochemical marker studies including CAM5.2, CK20, CK19, calponin, EMA, DOG-1, HER-2, estrogen receptor, and progesterone receptor showed nonreactive (negative) results. A CD117 study was nonreactive (negative) in the tumor but confirmed increased numbers of mast cells scattered exterior to the neoplastic epithelial nests within the intratumoral fibrous stroma.

## 3. Discussion

As is increasingly the case in large academic departments and other referral centers, the practice of anatomic pathology at the Cleveland Clinic is structured on a subspecialty model in which resected breast tissues are examined by pathologists who specialize in diagnoses of diseases of the breast, while skin specimens are studied by pathologists with focused training, experience, and interest in diseases of the skin. The specimen presented in this report was removed from the patient by a general surgeon and was submitted to the laboratory as an oriented excision labeled as “skin of the right breast.” The gross description, inking, and sectioning of the specimen were performed by a pathologist assistant, and the case was assigned to the breast pathology service. The pathologist of record (C.D.S.) recognized the tumor as a cutaneous adnexal neoplasm and sought consultation from a dermatopathology colleague (W.F.B.). The diagnosis of cylindroma was rendered on H&E stained histologic sections supplemented by an ancillary periodic acid-Schiff stain. While establishing this diagnosis was not particularly challenging in this well-oriented and completely resected tumor, it is possible that a smaller sampling of the tumor, such as a core biopsy, could have been misinterpreted as a basaloid-appearing primary breast neoplasm with a differential diagnosis potentially including basaloid forms of ductal carcinoma in situ, basaloid forms of solid/nested invasive ductal carcinoma, and adenoid cystic carcinoma. Breast cancers with basal and myoepithelial phenotypes are distinct groups of tumors that share some common morphological features and an association with poor prognosis [18]. The basal rather than the myoepithelial phenotype in breast carcinomas has the strongest relationship with patient outcome [18]. “Breast pathologists” should practice with an ever-present awareness that the target of their usual study is intimately wrapped in another potentially tumorigenic organ, the skin, and that the skin itself can give rise to both benign and malignant basaloid neoplasms.

Recent investigations regarding cylindroma tumorigenesis have confirmed that activation of MYB, in either wild-type forms or in fusion derivatives, is common in both spontaneous and hereditary cylindromas [19, 20]. MYB proteins are a family of transcription factors with highly conserved DNA binding domains that are involved in regulation of cellular differentiation and proliferation [21]. MYB driver mutations associated with oncogenesis have been confirmed in several types of malignancies including leukemias and adenoid cystic carcinomas [21]. Studies of familial kindreds with cylindroma syndromes have confirmed mutations in the* cylindromatosis* (CYLD) gene. This gene encodes an enzyme with deubiquitinase activity, and to date, more than 95 different disease-causing mutations have been published for the CYLD gene [22]. Most of these mutations are located in exons 9–20. Overexpression of MYB is known to drive proliferation of CYLD-defective cylindroma cells, and molecular heterogeneity in the pathogenesis of sporadic and inherited cutaneous cylindromas appears to converge on MYB activation [19]. Work comparing specific mutations in Brooke-Spiegler syndrome, familial cylindromatosis, and multiple familial trichoepithelioma type 1 kindreds may reveal genotype-phenotype correlations and may promote increased understanding of disease mechanisms allowing for development of future therapeutic modalities [22]. Targeted small molecule inhibitors of MYB transcription factors are currently being investigated as therapies for hematopoietic neoplasms, and characterization of these compounds suggests that disruption of protein-protein interactions of MYB and its coactivator p300 may be a suitable strategy to inhibit MYB driven tumorigenesis [23].

GATA3, mammaglobin, and E-cadherin immunohistochemical studies were performed on this cylindroma of the skin of the breast out of academic interest. GATA3 expression has been previously studied in epidermal and cutaneous adnexal neoplasms with published reports existing of variable positivity in cylindromas [24–26]. Focal mammaglobin immunoreactivity has also been previously reported in cutaneous cylindromas [27]. E-cadherin is known to be expressed on the cell surface of skin appendages [28]. It is important for practitioners of breast pathology to note that these commonly employed markers of the breast ductal epithelium are not specific for mammary ductal epithelium and/or tumors derived from mammary ductal epithelium, and all of these markers may be found positive in cylindromas of the skin of the breast. Of note, CK19, a marker expected positive in normal breast luminal epithelial cells and neoplasms derived from these cells, was found to be nonreactive (negative). Calponin, a marker expected positive in myoepithelial cells and some neoplasms derived from these cells, was also nonreactive (negative) in the tumor cells.

**Figure 6:**
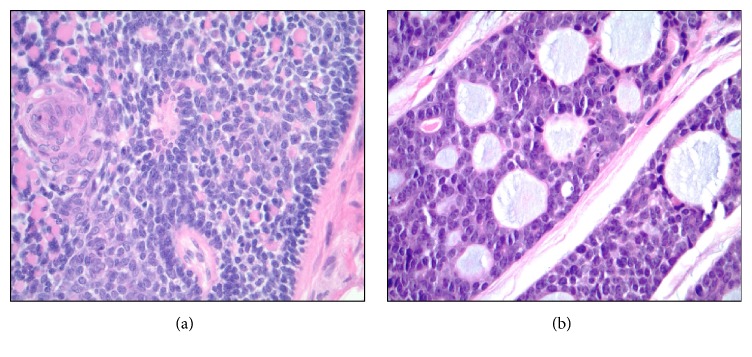
Histopathologic comparison of cylindroma with adenoid cystic carcinoma: (a) high magnification of cutaneous adnexal cylindroma of the breast showing small, dense, and eosinophilic cylinders of basement membrane material sprinkled throughout an epithelial island, H&E stain, 400x; (b) high magnification of a comparison case of mammary adenoid cystic carcinoma with a cribriform growth pattern and larger amphophilic accumulations of basement membrane material, H&E stain, 400x.

Adenoid cystic carcinoma of the breast is a rare special subtype of primary breast carcinoma characterized by the presence of a dual cell population of luminal and basaloid cells arranged in unique growth patterns [29, 30]. While adenoid cystic carcinomas of the breast generally display a basal-like phenotype and are most often “triple negative” (not expressing estrogen receptors, progesterone receptors, or HER-2), they behave in a more indolent fashion than other breast tumors with basaloid phenotypes [29]. Both dermatopathologists and breast pathologists can benefit from the recognition that there is a morphologic spectrum of primary salivary gland type tumor that can involve the breast and may extend into the skin of the breast [30]. Adenoid cystic carcinomas contain intrinsic basement membrane material, albeit in a different configuration than in cylindromas (Figure 6). Like cylindromas, adenoid cystic carcinomas have also been linked to MYB aberrations [31, 32]. The differential diagnosis of adenoid cystic carcinoma of the breast is an important one to remember in distinguishing cylindroma from malignancy with some authors emphasizing immunohistochemical staining differences (such as patterns of CK5/6 reactivity) [33]. Other investigators have suggested molecular techniques such as massively parallel sequencing for separating basaloid adenoid cystic carcinoma from breast cylindroma [34].

To our knowledge, fewer than 20 cases of cutaneous adnexal cylindroma of the breast have been reported in the world's literature. Our case adds to this list, and ancillary immunohistochemical marker studies performed out of academic interest highlight potential pitfalls with basaloid-appearing breast neoplasms. Recent investigations of MYB and SOX-10 expression in cutaneous adnexal tumors have shown 85% (17/20) immunohistochemical reactivity for both MYB and SOX-10 in cylindromas with SOX-10 studies showing more diffuse and intense staining patterns [35]. While SOX-10 is known to play an important role in melanocyte development and can be used as a diagnostic marker of malignant melanoma, this protein is less studied in tumors of the apocrine and eccrine sweat glands. Diffuse expression in cylindromas can be considered as a marker of myoepithelial differentiation [35]. While malignant transformation of cutaneous cylindromas is rare, reports of some cases do exist [36]. These rare examples of malignancy arising from cylindromas are seen more commonly in clinical settings of multiple tumors and may be associated with both locally destructive growth and metastases. Careful consideration of clinical setting, family history, imaging studies, and histopathologic details should allow for accurate diagnoses of cylindromas, even at unexpected body sites such as the breast. When necessary, special stains and immunohistochemical characterization may be of value in refining histopathologic diagnoses; however, it should be remembered that standard markers of mammary ductal epithelium such as GATA3 and mammaglobin may be expressed in basaloid cutaneous adnexal neoplasms.
